# Systematic review of prognostic models in traumatic brain injury

**DOI:** 10.1186/1472-6947-6-38

**Published:** 2006-11-14

**Authors:** Pablo Perel, Phil Edwards, Reinhard Wentz, Ian Roberts

**Affiliations:** 1 Nutrition and Public Health Intervention Research Unit, Epidemiology and Population Health Department, London School of Hygiene & Tropical Medicine, Keppel Street, London WC1E 7HT, UK

## Abstract

**Background:**

Traumatic brain injury (TBI) is a leading cause of death and disability world-wide. The ability to accurately predict patient outcome after TBI has an important role in clinical practice and research. Prognostic models are statistical models that combine two or more items of patient data to predict clinical outcome. They may improve predictions in TBI patients. Multiple prognostic models for TBI have accumulated for decades but none of them is widely used in clinical practice. The objective of this systematic review is to critically assess existing prognostic models for TBI

**Methods:**

Studies that combine at least two variables to predict any outcome in patients with TBI were searched in PUBMED and EMBASE. Two reviewers independently examined titles, abstracts and assessed whether each met the pre-defined inclusion criteria.

**Results:**

A total of 53 reports including 102 models were identified. Almost half (47%) were derived from adult patients. Three quarters of the models included less than 500 patients. Most of the models (93%) were from high income countries populations. Logistic regression was the most common analytical strategy to derived models (47%). In relation to the quality of the derivation models (n:66), only 15% reported less than 10% pf loss to follow-up, 68% did not justify the rationale to include the predictors, 11% conducted an external validation and only 19% of the logistic models presented the results in a clinically user-friendly way

**Conclusion:**

Prognostic models are frequently published but they are developed from small samples of patients, their methodological quality is poor and they are rarely validated on external populations. Furthermore, they are not clinically practical as they are not presented to physicians in a user-friendly way. Finally because only a few are developed using populations from low and middle income countries, where most of trauma occurs, the generalizability to these setting is limited.

## Background

Traumatic brain injury (TBI) is a leading cause of death and disability worldwide. Every year, an estimated 1.5 million people die and hundreds of millions require emergency treatment after a TBI. Fatality rates and disability rates vary depending on the severity and mechanisms of the TBI but unfavourable outcomes (death, vegetative state and severe disability) following TBI can be higher than 20%[1,2].

Physicians routinely make diagnostic and therapeutic decisions based on the patient's prognosis. Furthermore, prognostic information is also important in the counselling of patients and relatives in this critical scenario. Nevertheless in general, physicians believe that their predictions are inaccurate. A survey of doctors about prognosis in TBI found that only 37% thought that they currently assess prognosis accurately[3].

Prognostic models are statistical models that combine two or more items of patient data to predict clinical outcome. They may improve predictions in TBI patients. Some studies have shown that they are more reliable than what doctors can foretell [4]. A study conducted with TBI patients demonstrated that the introduction of a computer-based outcome prediction influenced patient management, with a higher use of resources in those patients with better prognosis [5].

Prognostic models could also be used in the design and analysis of Randomized Controlled Trials (RCTs). RCTs in TBI patients face many difficulties. Trauma is one of the most neglected research topics worldwide with a paucity of resources invested in RCTs [6]. Furthermore, unfamiliarity with issues of informed consent in unconscious patients pose further obstacles in this clinical setting [7]. Because of these barriers RCT in TBI are generally underpowered. A review of published RCTs in this area found that the average size was 82 participants per trial and no trial was large enough to detect reliably a 5% absolute reduction in risk [8]. Prognostic models have been proposed as a way to improve the power in TBI and stroke clinical trials [9,10]. With one such approach TBI patients' outcomes are defined taking account their baseline prognosis, instead of using the usual Glasgow Outcome Scale dichotomized in favourable or unfavourable.

Prognostic models can also assist in clinical audit by allowing adjustment for case-mix [11,12].

Multiple prognostic models for TBI have accumulated for decades but none of them is widely used in clinical practice. For a prognostic model to be clinically useful it should fulfil two requirements: it must be clinically valid and methodologically valid [13]. Systematic reviews of prognostic models in different areas of medical care have shown that models often fail in these two aspects [14,15]. Previous reviews of prognostic studies in TBI have only focused on individual predictors or have been restricted to prognostic models of some type of traumatic brain injury or outcome. So far, there has not been any comprehensive systematic review of prognostic models in traumatic brain injury [16,17]. It has then become increasingly important to identify and evaluate prognostic models in TBI patients.

### Objective

Our objectives were

(a) identify prognostic models in traumatic brain injury

(b) describe their characteristics

(c) investigate their quality and

(d) described the models that were validated in an external population.

## Methods

### Type of studies

We included studies that gave an overall prognostic estimation combining the predictive information from at least two variables. Studies could develop new prognostic models (derivation studies) or evaluate previous ones (validation studies). Studies conducted prior to 1990 were excluded because patient management and diagnostic techniques may have changed since this time. Studies that investigate more than one variable but do not combine them for obtaining a prediction were excluded.

### Type of exposures

Only variables that were collected before hospital discharge were considered as predictors. Glasgow Coma Scale (GCS) was considered as one predictor variable.

### Type of participants

Patients of any age with any type or severity of traumatic brain injury.

### Type of outcome measures

Studies that predict any outcome in traumatic brain injury patient (i.e. neurological impairment, disability, survival, etc.). There was no time restriction for the evaluation of the outcomes.

### Search strategy for identification of studies [see Additional file 1]

The reference lists of included studies were inspected for further possible studies meeting the inclusion criteria. A forward search (citing references in the Web of Knowledge) was conducted with selected seminal papers and some of the citing papers, not found by the database search, were inspected for relevance and possible inclusion. All records were converted into an Endnote database.

### Trial identification and selection

Two reviewers (PP & PE) independently examined titles, abstracts and keywords of records from electronic databases, for eligibility. The full text of all potentially relevant records was obtained and two reviewers (PP & PE) independently assessed whether each met the pre-defined inclusion criteria. Disagreement was resolved by a third reviewer (IR).

### Quality assessment

Quality assessment scores for controlled clinical trials and diagnostic studies have been criticized [18,19]. The main problem with quality scores is to determine the weight that each item should provide to the overall score. The abundance of quality scores shows that there is no consensus on this issue. Instead, a component approach appraisal allows one to evaluate each methodological aspect. Depending on the question and the study design some components may be more relevant than others (e.g. with a surgical intervention blinding of the patient and caregiver would be unachievable)

In studies of prognostic models in particular, although diverse quality assessment criteria have been proposed, there is none widely accepted [14,20-22]. We analyzed the quality of the prognostic models included in this systematic review considering two main domains:

a) Internal validity. This refers to the systematic error of the study and is related to study design, variables and analysis strategy.

b) External validity or generalizability. This refers to the extrapolation of the study to other settings. For making judgments about generalisability it is important to consider the characteristics of the sample from which the model was derived, the clear presentation of the results and finally the model should, ideally, be evaluated (validated) in a different sample from the original.

Taking into account these two domains,18 questions were considered for each of the models included [see Additional file 2].

We restricted the quality assessment to the derivation studies.

### Performance of models externally validated

We reported the performance of models that were validated in an external sample. We considered as externally validated those models that were reported by the authors as evaluated in a different cohort of TBI patients from the derivation set.

### Data extraction

One reviewer (PP) extracted the information from each study for assessing the quality of reporting in each of the questions.

## Results

**Figure 1:**
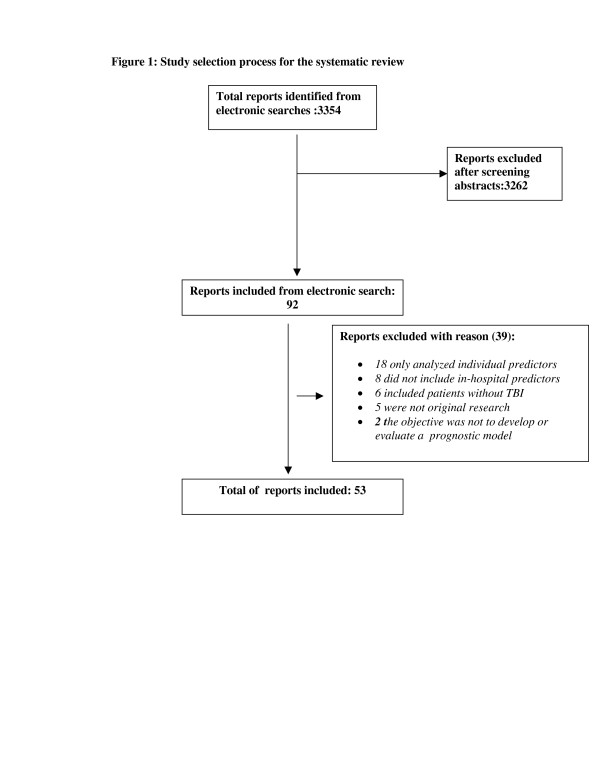
Study selection process for the systematic review of prognostic models in head injury.

A total of 3354 records were identified. After reading all the records 92 reports were identified and read in full. Thirty nine were excluded for the following reasons: 18 analyzed individual predictors but did not combine them in a single score, eight did not include in-hospital predictors, six included patients without traumatic brain injury, five were not original research (e.g. discussion, letter) and in two the objective was not to evaluate prognosis in TBI patients. (Figure 1)

The remaining 53 reports described 102 prognostic models [see Additional file 3].

### General characteristics of the prognostic models [see Additional file 4]

#### Population included

Almost half (47%) of the models were derived from an adult population, 12% were derived from a child population while 21% were derived from a population that included both adults and children. In 21% of the models it was not clearly reported from which population they were derived.

In relation to the severity of the TBI studied, forty five models (44%) included all grades of severity, thirty one (30%) included severe TBI, nine (9%) moderate or severe TBI, nine (9%) mild TBI and in eight (8%) the severity of TBI was not clearly reported.

A median of 319 patients (range 22–7764 patients) were included per model. Three quarters included less than 500 patients.

A total of ninety five models (93%) included populations from high income countries, five (5%) included populations from middle income countries and in two (2%) the population was from a low income country.

### Objectives

Most of the models reported (65%), were derived for the first time (derivation models) while in 35% the models reported were validating pre-existing models (validation models). The majority of the validation models (29 out of 35) validated general trauma score. The remaining 6 models validated specific TBI prognostic score. One validation model was reported as a letter and information was limited. Three models validated prognostic scores that were developed before 1990.

### Variables included as predictors

A total of 89 variables were included in the prognostic models. A mean of 5 variables were included in each model (range 2 to 13). GCS was the most common predictor included in the models,(50%) followed by age (46%) and pupil reactivity (26%). Overall clinical variables were included in 66% of the models, demographic variables were included as predictors in 50% of the models, CT scan predictors were used in 19% of the models and 7% included variables related to characteristics of the injury. In 7% of the models other predictors were included (e.g. other complementary tests or existing scores).

### Outcomes

Mortality was the main outcome in 30% of the models and GOS in 28%. Other functional outcomes were reported in 31% of the models. The presence of a CT scan lesion was the main outcome in 7%, the need of neurosurgical intervention in 2% and raised intracranial pressure in 1%.

### Analysis

In the multivariate analysis for the development of prognostic models (n:66) logistic regression was used in 31 (47%) models. Regression tree analysis was reported in 14 (21%) and neural networks in nine (13%). Other methods of analysis were performed in nine (14%) models while in one (2%) it was not clear and in three (5%) no multivariable analysis was performed.

### Quality assessment (table 1)

**Table 1:** Quality assessment of prognostic models

**INTERNAL VALIDITY**	*All models*	*Logistic regression*	*Other analysis*
	*N:66*	*N:31*	*N:35*
**STUDY**			
Loss to follow-up			
< 10%	10 (15%)	5 (16%)	5 (14%)
>10%	19 (29%)	7 (23%)	12 (34%)
Not reported	37 (56%)	19(61%)	18 (52%)
**VARIABLES**			
Discussion about predictors			
Yes	21(32%)	11 (35%)	10(29%)
No	45(68%)	20 (65%)	25 (71%)
Description of measurement of predictors			
Yes	12 (18%)	8 (26%)	3 (9%)
No	54 (82%)	23(74%)	32 (91%)
Validity of outcome reported			
Yes	31 (47%)	14 (45%)	17(49%)
No	20 (30%)	7 (23%)	13 (37%)
Not applicable	15(23%)	10 (32%)	5 (14%)
Handling of missing data			
Estimated statistically	4 (6%)	4 (13%)	0
Excluded	36(55%)	16(52%)	20 (57%)
Not reported	26(39%)	11(35%)	15 (43%)
**ANALYSIS**			
Multivariable analysis Stepwise			
Backwards	-	12 (39%)	N/A
Forwards	-	3 (10%)	
Not specified	-	10 (32%)	
Not reported	-	5 (16%)	
Other	-	1 (3%)	
Interactions examined			
Yes	-	4 (13%)	
Not reported	-	27 (87%)	
Handling of predictors variables			
Continuous	-	6 (19%)	
Categorical	-	16 (52%)	
Not clear	-	9 (29%)	
More than 10 events per variable			
Yes	-	9 (29%)	
No	-	16 (52%)	
Not reported	-	6 (19%)	
**EXTERNAL VALIDITY**	All models	Logistic regression	Other analysis
Description of the sample			
Yes	55 (83%)	28 (90%)	27 (77%)
No	11(17%)	3 (10%)	8 (23%)
Presentation of the prognostic model			
Normogram	1 (1%)	1 (3%)	0
Simplified score	8 (12%)	4 (13%)	4 (11%)
Figure	13 (20%)	1 (3%)	12 (34%)
Regression formula	15 (23%)	12(39%)	3 (9%)
Not explained	29 (44%)	13(42%)	16 (46%)

**EXTERNAL VALIDITY**	*All models*	*Logistic regression*	*Other analysis*
	*N:66*	*N:31*	*N:35*

Performance reported A.U.C (Discrimination)			
Yes	-	18 (58%)	NA
No	-	13(42%)	
C.I. presented	-	8 out of 18 (44%)	
H-L (Calibration)			
Yes	-	7 (23%)	NA
No	-	23(74%)	
Other	-	1 (3%)	
Overall accuracy			
Yes	37 (56%)	15 (48%)	22 (63%)
No	29 (44%)	16 (52%)	13 (17%)
Validation			
Yes	25 (38%)	17 (55%)	8 (23%)
*External*	*7 (11%)*	*7 (23%)*	*0*
No	41 (62%)	14 (45%)	27 (77%)

We restricted the quality assessment to the 66 derivation models. Some of the quality assessment items could only be applied to logistic regression models.

### Internal validity

In over half of the models loss to follow-up was not reported;15% reported an adequate loss to follow-up (less than 10%).

Most of the models (68%) did not include a discussion about the rationale to include the predictors in the model. A detailed description of the measurement of the predictors was absent in 82% of the models. In one third of the models the validity of the outcome measures was not reported.

In relation to the analysis of those that used multivariate logistic regression, stepwise was the most common approach (81%). Interactions were examined in 13% of the models. Predictor variables were analyzed as continuous in 19% of the models. A third (29%) of the models included at least 10 events per variable analyzed as predictor. The most common strategy to handle missing data was exclusion of observations (55%).

### External Validity

The sample was described in almost all the models (83%). The procedure to obtain the score was explained in approximately half of the models (56%), however in those that used logistic regression only 19% included a user-friendly presentation.

In relation to the performance of the models, discrimination was reported in 58% of the models through the area under the receive operator curve (A.U.R.O.C.), 44% of which included the respective confidence interval. Calibration was reported with the Homer-Lemeshow test in 27% of the models. Almost half the models(56%) reported their overall accuracy.

Less than half of the models (38%) were validated, of which 11% were validated in an external population.

None of the models was evaluated prospectively in a randomized clinical trial to assess the effect in clinical practice.

### Description of externally validated models

Seven models were developed and also reported an external validation (table 2). Two other models were validation of pre-existing models.

**Table 2:** Characteristics of models externally validated

**Author**	**Derivation sample**	**Validation sample**	**Predictors**	**Outcomes**	**Performance in the validation sample**	**Presentation of a simplified score**
Pillai et al.	208 patients from India with severe TBI	26 patients from the same centre	1-oculocephalic reflex 2-motor GCS 3-midline shift	Death or vegetative state	Sensitivity (75%) Specificity (67%) PPV 50%	No
Signorini et al	372 patients from Scotland with moderate and severe TBI	520 patients from the same centre	1-GCS 2-ISS 3-pupils reactivity and 4-haematoma (CT scan)	Survival at 1 year	A.U.R.O.C (0.835) Error rate (15.2%) Brier score (0.1160) Hosmer-Lemeshow (p < 0.001)	Nomogram
Signorini et al	110 patients from Scotland with moderate and severe TBI	140 patients from the same centre	1-GCS 2-ISS 3-pupils reactivity and 4-haematoma (CT scan) 5-ICP measures	Survival at 1 year	Not reported	No
Hukkelhoven et al.	134 patients from Netherlands with moderate and severe TBI	180 patients from the same centre	1-age 2-motor GCS 3-pupils reactivity 4-pupillary size 5-hypotension 6-ISS	Raised ICP	A.U.R.O.C. (0.50) Hosmer-Lemeshow (p = 0.18)	No
Hukkelhoven et al.	275 patients from Netherlands with moderate and severe TBI	250 patients from the same centre	1-age 2-cause of injury 3-pupils reactivity 4-pupillary size 5-hypotension 6-ISS	Surgical removable lesions	A.U.R.O.C. (0.67) Hosmer-Lemeshow (p = 0.01)	No
Hukkelhoven et al.	2269 patients from 2 trials in high income countries with moderate and severe TBI	796 patients from Europe	1-age 2-motorGCS 3-pupils reactivity 4-hypoxia 5-hypotension 6-CT classification 7-subarachnoid haemorrhage	Death or disability at 6 months	A.U.R.O.C. (0.83) Hosmer-Lemeshow (p = 0.05)	Score chart
Hukkelhoven et al.	2269 patients from 2 trials in high income countries with moderate and severe TBI	796 patients from Europe and 746 from the United States	1-age 2-motor GCS 3-pupils reactivity 4-hypoxia 5-hypotension 6-CT classification 7-subarachnoid haemorrhage	Death at 6 months	A.U.R.O.C. (0.87/0.89) Hosmer-Lemeshow (p = 0.42/<0.001)	Score chart

Pillai *et al. *developed a prognostic model to predict unfavourable outcome (death or vegetative state) at one month [23]. They developed the model in a cohort of 289 patients and validated the model in 26 patients from the same centre. The predictor variables were oculocephalic reflex, motor score of the GCS and midline shift score. In the validation set they reported sensitivity (75%), specificity (67%), predictive value of unfavourable outcome (50%), predictive value of favourable outcome (86%), percentage of false optimistic results (25%), and percentage of false pessimistic results (33%). They did not report the model's performance measured in the derivation set. Confidence intervals of the estimates were not reported. Although the authors reported how to calculate the prediction score, they did not present it in a user-friendly fashion.

Signorini *et al. *developed two prognostic models, for one they used only clinical variables and for the other they added variables on secondary insults. [24,25] In both models the outcome was survival at 1 year. The first model was validated in 520 patients who attended the same centre. The predictors were age, GCS, ISS, pupils reactivity and presence of haematoma on the CT scan. They reported measures of discrimination: A.U.R.O.C. (0.835), error rate (15.2%) and calibration: brier score (0.1160), Hosmer-Lemeshow (p < 0.0001). They included a graph with the 95% confidence interval of the calibration of the model. The second model was validated in 140 patients who attended the same centre. The predictor variables were the same as the first model plus ICP measures. Although they mentioned that brier score, error rate, A.U.R.O.C were higher than the original dataset they did not report the actual estimates. They reported a normogram to predict probability of survival that is user-friendly for physicians.

Hukkelhoven *et al. *reported four different models [26,27]. The outcomes were: raised intracranial pressure (ICP), surgically removable lesions (SRL), unfavourable outcome (death, vegetative state or severe disability) and mortality at six months. For the validation of the first two outcomes they use an historical (previous) sample of 205 patients from the same centre. The predictors for ICP were age, motor score, pupil size, pupillary reactivity, hypotension and ISS. For SRL the predictors were the same except for motor score which was not, and cause of injury that was added. For unfavourable outcome they used one database and for mortality two databases, none of these databases were related with the population of the derivation set. The predictor variables were age, gender, cause of injury, pupil reactivity, hypotension, hypoxia, CT classification and traumatic subarchnoid haemorrhage They reported the models discrimination: A.U.R.O.C. of 0.50 (95% CI 0.41–0.58), 0.67 (95% CI 0.60–0.75) and 0.83 (95% CI 0.80–0.86) for ICP, SRL, unfavourable outcome and mortality respectively. They also reported the model calibration: Hosmer-Lemeshow goodness of fit test of 0.18, 0.01,0.05 and 0.42 (<0.001), for ICP, SRL, unfavourable outcome and mortality respectively (the calibration of the mortality model was validated in two different databases). They presented the model as a score chart to facilitate its use in clinical practice.

Bush *et al. *validated a model previously developed by the same group [28]. Their model was intended to allow better understanding of factors influencing functional outcomes and was not intended to predict individual outcomes. It was not clearly reported whether the patients came from the same original population. They used path analysis to evaluate the predictors (functional status, injury severity and cognitive status) on functional outcomes (disability rating scale, community integration questionnaire and return to employment). The reported difference indexes of goodness of fit that showed that the originally model fitted better than the validation model. They did not report any discrimination measures.

Benzer *et al. *validated a model that used an existing scale, although they did not provide details of when and how it was developed [29]. They did not use any kind of multivariable analysis. The used a score based in the following variables : reaction to acoustic stimuli, reaction to pain, body posture, eye opening, pupil size, pupil response to light, position and movements of eyeballs and oral automatisms to predict mortality at 21 days. They did not report any performance measure, but just the chi square test for survival of those with low versus high score. They presented the score in a user-friendly way.

## Discussion

This systematic review shows that although publications of prognostic models for TBI patients are very frequent their quality is relatively poor. In addition they are rarely validated on external populations or presented to physicians in a friendly way. Furthermore, only a few are developed using populations from low and middle income countries where most trauma occurs.

Patients from all severity spectra were investigated but prognostic models for moderate and severe TBI patients were more frequent. It is noteworthy that only 2% of the models included patients from low income countries taking into account that 90% of trauma occur in these countries. Although biologically prognostic factors should be the same worldwide, is reasonable to consider that the strength of the association could differ depending on the medical care received. This difference could affect the accuracy of the prognostic models in different settings. Although there is no data about this, an ongoing project analysing the MRC CRASH Trial Cohort is exploring this issue.

GCS, age and pupil reactivity were the most common variables analyzed as predictors whereas, G.O.S. and mortality were the most common outcomes investigated. Multiple logistic regression was the multivariable analysis most frequently used.

We found several limitations in the quality of the models. The majority did not include a thorough discussion of the rationale for including the predictor variables. Only a minority had a loss to follow-up of less than 10%. This is potentially an important limitation as the loss to follow-up could be related to prognosis and this could lead to biased results. Furthermore only four models handle appropriately the missing data with statistical imputation. In relation to the multivariable analysis, automatic procedures (stepwise) were quite common in logistic regression. There is no agreement in relation to the appropriateness of this strategy. This is shown, for example, in conflict recommendations in quality assessment for prognostic studies; while in one study the use of stepwise was considered as good quality in other it was considered as an indicator of a fatal flaw [30]. One of the limitations we found was that most of the studies did not explicitly consider clinical criteria to enter the variables in the model beyond the automatic procedures. Interactions were hardly ever explored although this is strongly recommended in multivariable analysis [31]. Another common weakness in the logistic regression models was the lack of power of the models, as only one third included at least 10 events per variable. It has been proposed that this is the minimum ratio of events to variables which is large enough to allow an adequate precision of the estimates [31].

We did not attempt to obtain an overall quality assessment and instead we evaluated its different components, this approach makes a cross comparison between different analytical strategies difficult because, for example many of the criteria only apply to logistic regression analysis.

It is also important to report how well the model works and for this performance measures should be reported. Remarkably only two thirds reported a measure of discrimination and only one fifth a measure of calibration. This is of particular concern considering that calibration is the most important performance measure for the application of the models in clinical practice [32]. Even when a discrimination measure was reported, less than half presented confidence intervals to provide readers an estimation of the precision.

For a model to be generalizable to other populations it is very important to conduct an external validation [32]. Only seven models (three reports) developed and validated a model but in only one of them the validation was performed on patients of a different centre. Those models that considered mortality as an outcome found A.U.R.O.C that were higher than 0.70 which is considered as excellent discrimination. However the discrimination for the other outcomes were not as good. Furthermore, the calibration measures were low in all the outcomes considered.

Finally, to be useful, the method to estimate prognosis should be clearly reported and, to be clinically practical, they should be user-friendly. In only half of the models was it clearly explained how to obtain the prognostic score and in only one tenth was it reported in such a way that could be easily applicable in a clinical setting.

From all of the models found in our systematic review we consider that those developed by Hukkelhoven et al. and Signorini et al. are the most clinical useful for patients from high income countries with moderate and severe TBI, as they fulfilled the majority of the methodological requirements and showed an acceptable performance in the external validation, furthermore they are available in a user-friendly way [27,25].

We acknowledge some limitations in our study. Firstly, we only included studies that explicitly combined at least two predictors, in doing so we could have missed some reports that used multivariable to analyze individual predictors and did not report in the abstract the overall estimation although they included the estimate in the full report. Secondly, we did not include studies that assessed clinical predictor rules for which although they considered more than one variable they did not combine them. We considered that the methodological framework to assess such studies is fundamentally different from prognostic models. Thirdly, we restricted our search to 1990 onwards so, we could have missed some relevant prognostic models published prior to that date. However because of changes in management and diagnostic technology in recent years we doubt that prognostic models previous to 1990 could be useful for the current medical care of TBI patients. Finally, another limitation of this paper is that we did not describe the time of prediction assessment of the prognostic models. Although we acknowledge that this information can be clinically very useful unfortunately this data was seldom available in the reports.

To our knowledge there has been only one previous systematic review of prognostic models in TBI [17]. They found 10 reports, all of which were identified in our systematic review. They validated four of these reports (6 models) in four series of patients. Discrimination(A.U.R.O.C.) in the validation series ranged from 0.70 to 0.80. On the other hand calibration was poor. They concluded that large sample sizes and refitting of the original model coefficients are related with a better performance of the models. Unlike ours this systematic review was restricted to models that use baseline characteristics to predict mortality or unfavourable outcome (defined by G.O.S.) in moderate and severe TBI patients. Furthermore the search strategy was not specified.

Systematic reviews of prognostic models for other diseases have found similar results to ours. For example Counsel *et al. *conducted a systematic review of prognostic models in patients with acute stroke [14]. They found 83 prognostic models but they concluded that none of them has been sufficiently well developed and validated.

## Conclusion

This systematic review describes the limitations of published prognostic models in TBI and most importantly inform researchers who are involved in the development of prognostic models in TBI. Future studies should consider the following issues to develop valid prognostic models: thorough discussion with physicians of potential predictors that are "clinically relevant", clear description of the measurement and validity of variables included in the model, large sample size to ensure precise estimates, adequate handling of continuous variables and missing data, assessment of interaction in the multivariable analysis, clear description of the calculation of the prognostic score, external validation and adequate report of model performance measures, such that physicians can interpret their accuracy. It should also be encouraged that more studies include population from low and middle income countries where most of the burden of TBI occurs. Finally, for prognostic models to be clinical useful they should be presented in user-friendly way to be easily applied in the clinical scenario.

## Competing interests

The author(s) declare that they have no competing interests.

## Authors' contributions

PP, IR and PE developed the protocol and conducted the SR. RW conducted the searching. PP conducted the analysis. All the authors read and approved the final manuscript.

## Pre-publication history

The pre-publication history for this paper can be accessed here:



## Supplementary Material

Additional File 1 Electronic bibliographical databases and search strategies. This table describe the databases and search strategies used in the systematic review. Click here for file

Additional File 2 Quality assessment of prognostic models. This document describe the different items analysed for the quality assessment. Click here for file

Additional File 3 Studies included in the systematic review. List of references of included studies in the systematic review Click here for file

Additional File 4 General Characteristics of the models. This table describe the characteristics of the included models in the systematic review Click here for file
