# Supplementary material for: Systematic review of prognostic models in traumatic brain injury
Source: BMC Med Inform Decis Mak. 2006 Nov 14;6:38. doi: 10.1186/1472-6947-6-38 (PMC1657003; doi:10.1186/1472-6947-6-38)
Supplement: Additional File 1 — Electronic bibliographical databases and search strategies. This table describe the databases and search strategies used in the systematic review. [file 1472-6947-6-38-S1.doc]

*Electronic bibliographical databases and search strategies*

| **Database (time period or version)** | **Search Strategy** |
| --- | --- |
| Medline(PUBMED version)  limit to 1990 – 2005 | [brain injuries OR traumatic brain injury OR craniocerebral trauma] OR [ "brain injuries" OR "Traumatic brain injury " OR "brain Trauma" OR "brain Trauma" Field: Title] AND [ brain[ti] OR brain*[ti] OR coma[ti] OR conscious*[ti] OR cranio*[ti] OR skull[ti]] AND ["Case-Control Studies"[MeSH] OR "Cohort Studies"[MeSH] OR "Follow-Up Studies"[MeSH] OR prognos* OR predict* Field: Title] |
| Embase(OVID version):  yr=1990-2005 | 1. traumatic brain injury .mp. or exp traumatic brain injury / or exp *traumatic brain injury / or brain injur$.ti. 2. (brain$ or brain$ or coma$ or conscious$ or cranio$ or skull$).ti. 3. 1 and 2  4. (prognos$ or predict$).mp. 5. 3 and 4 6. case control study.mp. or (cohort study or cohort analysis).mp. or exp follow up/ or exp case control study/ or follow up.mp. or systematic review.mp. or trial.mp. or randomi$.mp. 7. 5 and 6  8. limit 7 to |
