# Supplementary material for: Systematic review of prognostic models in traumatic brain injury
Source: BMC Med Inform Decis Mak. 2006 Nov 14;6:38. doi: 10.1186/1472-6947-6-38 (PMC1657003; doi:10.1186/1472-6947-6-38)
Supplement: Additional File 2 — Quality assessment of prognostic models. This document describe the different items analysed for the quality assessment. [file 1472-6947-6-38-S2.doc]

**Quality assessment of prognostic models**

**Internal validity**

***Study***

**1) Did the patients have an adequate follow-up?** *In the case of prognosis in TBI all the studies are conceptually cohort studies, although the method of data collection could be either prospective or retrospective (i.e. use of databases). In cohort studies a large loss to follow-up could lead to attrition bias. To minimize attrition bias the follow-up should be at least 90 % of the original cohort*

***Variables***

**2) Was a discussion included about rationale to include the predictors?** *The variables included should be important predictors reported in previous studies. (e.g for early indicators in severe traumatic brain injury the systematic review conducted by the Brain Trauma Foundation identified five: Glasgow Coma Scale (GCS), age, pupillary reflex, hypotension, CT scan features)*

**3) Were the predictive variables clearly defined?** *(variables definition and measurement should be clearly described in the method section of the report)*

**4) Were the outcomes predicted valid ?** *(GOS is the most frequent outcome considered in TBI studies and its validity has been reported previously. If other outcomes are considered, validity should be reported unless mortality is the outcome for which validity is not applicable )*

**5) Were missing data adequately managed?** *(Imputation strategies are preferable to complete case analysis)*

***Analysis***

**6) Was an adequate strategy performed to build the multivariable model?** (*Multivariable analysis strategy should consider clinical criteria when entering variables in the model and not only automatic selection strategies such as stepwise where, in some cases important clinical predictors could be “forced” into the model)*

**7) Were interactions between the variables examined?** *(when a multivariable analysis is performed interactions between variables should be explored)*

**8) Were continuous variables handled appropriately?** (*It is preferable to keep them as originally recorded because they can give more information and are more powerful to detect an association. Categorization of a variable in two groups assumes a constant risk in two of the each group created, which is often not true.* *In the case where variables are categorized, the rationale for the cut-off should be clearly explained)*

**9) Were more than 10 events per variable included?** *(The estimates will be unreliable if the data contain less than 10 outcome events relative to the number of predictors)*

**External validity or generalizability**

**10) Was the description of the sample reported?** *(For making judgment about generalizability it is important to know the characteristics of the sample from which the model was derived, so it is very important that studies include information about the population included: e.g. time of inclusion of the patients in relation to the injury, time of the measurement of the variables, treatment received)*

**11) Was it clearly explained how to estimate the prognosis?** *(For a prognostic model to be clinically useful it should be clearly explained how to estimate the prognosis in a clinical setting. Probability of the outcome could be obtain through simple scores, normograms or simple figures. Reporting just the coefficients of the multivariable model is not enough to be clinical practical in the emergency setting)*

**12) Were measures of discrimination reported?** *(To evaluate a model performance we should assess discrimination and calibration: Discrimination refers to the ability to rank in the correct order individuals with different prognosis. It is usually measured with the area under the Receiving Operator Curve (A.U.R.O.C) and is most important if prediction is used to stratify a group of patients by their risk to compare an intervention.)*

**13) Were measures of calibration reported?** *(For assessing the usefulness of a model the calibration should be referred. Calibration refers to the ability to predict correctly the prognosis-not to high or too low-. It could be measured graphically or with the Hosmer-Lemeshow test)*

**14) Were confidence intervals presented?** *(Clinicians that will use the model should know the precision of the estimates derived from the model)*

**15) Was the model validated?** *(For a prognostic model to be generalized in a population different from the one from which the model was derived it should be evaluated –validated- in a different set of patients)*

**16) Was the model internally validated?** *(Refers to the evaluation of the model in a sample of patients that were not included in the derivation set but are from the same population, it is evaluated with resampling methods, such as bootstrapping.)*

**17) Was the model externally validated?** *(Refers to the evaluation of the model in a different population (i.e. different geographical region, historical periods or different methods of data collection)*

**18) Was the effect of using the model established?** *(If the model was developed to use in clinical practice it should be evaluated with a randomized clinical trial)*
