# Supplementary material for: Systematic review of prognostic models in traumatic brain injury
Source: BMC Med Inform Decis Mak. 2006 Nov 14;6:38. doi: 10.1186/1472-6947-6-38 (PMC1657003; doi:10.1186/1472-6947-6-38)
Supplement: Additional File 3 — Studies included in the systematic review. List of references of included studies in the systematic review [file 1472-6947-6-38-S3.doc]

**Studies included in the systematic review**

1. Hukkelhoven CW, Steyerberg EW, Habbema JD, et al. Admission of patients with severe and moderate traumatic brain injury to specialized ICU facilities: a search for triage criteria. *Intensive Care Med* 2005;**31**(6)**:**799-806.

2. Hukkelhoven CW, Steyerberg EW, Habbema JD, et al. Predicting outcome after traumatic brain injury: development and validation of a prognostic score based on admission characteristics. *J Neurotrauma* 2005;**22**(10)**:**1025-39.

3. Hsu MH, Li YC, Chiu WT, et al. Outcome prediction after moderate and severe head injury using an artificial neural network. *Stud Health Technol Inform* 2005;**116:**241-5.

4. Poon WS, Zhu XL, Ng SC, et al. Predicting one year clinical outcome in traumatic brain injury (TBI) at the beginning of rehabilitation. *Acta Neurochir Suppl* 2005;**93:**207-8.

5. Wechsler B, Kim H, Gallagher PR, et al. Functional status after childhood traumatic brain injury. *J Trauma* 2005;**58**(5)**:**940-9; discussion 950.

6. Levin HS, McCauley SR, Josic CP, et al. Predicting depression following mild traumatic brain injury. *Arch Gen Psychiatry* 2005;**62**(5)**:**523-8.

7. Carter BG, Butt W. A prospective study of outcome predictors after severe brain injury in children. *Intensive Care Med* 2005;**31**(6)**:**840-5.

8. Eftekhar B, Mohammad K, Ardebili HE, et al.. Comparison of artificial neural network and logistic regression models for prediction of mortality in head trauma based on initial clinical data. *BMC Med Inform Decis Mak* 2005;**5**(1)**:**3.

9. Rovlias A, Kotsou S. Classification and regression tree for prediction of outcome after severe head injury using simple clinical and laboratory variables. *J Neurotrauma* 2004;**21**(7)**:**886-93.

10. Demetriades D, Kuncir E, Murray J, et al. Mortality prediction of head Abbreviated Injury Score and Glasgow Coma Scale: analysis of 7,764 head injuries. *J Am Coll Surg* 2004;**199**(2)**:**216-22.

11. Ibanez J, Arikan F, Pedraza S, et al. Reliability of clinical guidelines in the detection of patients at risk following mild head injury: results of a prospective study. *J Neurosurg* 2004;**100**(5)**:**825-34.

12. Fabbri A, Servadei F, Marchesini G, et al. Prospective validation of a proposal for diagnosis and management of patients attending the emergency department for mild head injury. *J Neurol Neurosurg Psychiatry* 2004;**75**(3)**:**410-6.

13. Tender GC, Awasthi D. Risk stratification in mild head injury patients: the head injury predictive index. *J La State Med Soc* 2003;**155**(6)**:**338-42.

14. Bush BA, Novack TA, Malec JF, et al. Validation of a model for evaluating outcome after traumatic brain injury. *Arch Phys Med Rehabil* 2003;**84**(12)**:**1803-7.

15. Pillai SV, Kolluri VR, Praharaj SS. Outcome prediction model for severe diffuse brain injuries: development and evaluation. *Neurol India* 2003;**51**(3)**:**345-9.

16. Brenner T, Freier MC, Holshouser BA, et al. Predicting neuropsychologic outcome after traumatic brain injury in children. *Pediatr Neurol* 2003;**28**(2)**:**104-14.

17. Cassidy LD, Potoka DA, Adelson PD, et al. Development of a novel method to predict disability after head trauma in children. *J Pediatr Surg* 2003;**38**(3)**:**482-5.

18. Ratanalert S, Chompikul J, Hirunpat S, et al. Prognosis of severe head injury: an experience in Thailand. *Br J Neurosurg* 2002;**16**(5)**:**487-93.

19. Andrews PJ, Sleeman DH, Statham PF, et al. Predicting recovery in patients suffering from traumatic brain injury by using admission variables and physiological data: a comparison between decision tree analysis and logistic regression. *J Neurosurg* 2002;**97**(2)**:**326-36.

20. Heard C, Li V, Heard A. A useful tool for predicting outcome for the pediatric head trauma patient. *Crit Care Med* 2002;**30**(6)**:**1403-4.

21. Schaan M, Jaksche H, Boszczyk B. Predictors of outcome in head injury: proposal of a new scaling system. *J Trauma* 2002;**52**(4)**:**667-74.

22. Schreiber MA, Aoki N, Scott BG, et al. Determinants of mortality in patients with severe blunt head injury. *Arch Surg* 2002;**137**(3)**:**285-90.

23. Sustic A, Turina D, Ticac Z, et al. War head injury score: an outcome prediction model in War casualties with acute penetrating head injury. *Mil Med* 2001;**166**(4)**:**331-4.

24. Sinha M, Kennedy CS, Ramundo ML. Artificial neural network predicts CT scan abnormalities in pediatric patients with closed head injury. *J Trauma* 2001;**50**(2)**:**308-12.

25. Mukherjee KK, Sharma BS, Ramanathan SM, et al.. A mathematical outcome prediction model in severe head injury: a pilot study. *Neurol India* 2000;**48**(1)**:**43-8.

26. Ashwal S, Holshouser BA, Shu SK, et al. Predictive value of proton magnetic resonance spectroscopy in pediatric closed head injury. *Pediatr Neurol* 2000;**23**(2)**:**114-25.

27. Wagner AK, Hammond FM, Sasser HC, et al. Use of injury severity variables in determining disability and community integration after traumatic brain injury. *J Trauma* 2000;**49**(3)**:**411-9.

28. Vath A, Meixensberger J, Dings J, et al. Prognostic significance of advanced neuromonitoring after traumatic brain injury using neural networks. *Zentralbl Neurochir* 2000;**61**(1)**:**2-6.

29. Adachi S, Hirano N, Tanabe M, et al. Multivariate analysis of patients with head injury using quantification theory type II--with special reference to prediction of patient outcome. *Neurol Med Chir (Tokyo)* 2000;**40**(4)**:**200-4; discussion 204-5.

30. Wagner AK, Hammond FM, Grigsby JH, et al. The value of trauma scores: predicting discharge after traumatic brain injury. *Am J Phys Med Rehabil* 2000;**79**(3)**:**235-42.

31. Stuss DT, Binns MA, Carruth FG, et al. Prediction of recovery of continuous memory after traumatic brain injury. *Neurology* 2000;**54**(6)**:**1337-44.

32. Lannoo E, Van Rietvelde F, Colardyn F, et al. Early predictors of mortality and morbidity after severe closed head injury. *J Neurotrauma*. 2000;**17**(5):403-14.

33. Nissen JJ, Jones PA, Signorini DF, et al. Glasgow head injury outcome prediction program: an independent assessment. *J Neurol Neurosurg Psychiatry* 1999;**67**(6)**:**796-9.

34. Sakellaropoulos GC, Nikiforidis GC. Development of a Bayesian Network for the prognosis of head injuries using graphical model selection techniques. *Methods Inf Med* 1999;**38**(1)**:**37-42.

35. Signorini DF, Andrews PJ, Jones PA, et al.. Adding insult to injury: the prognostic value of early secondary insults for survival after traumatic brain injury. *J Neurol Neurosurg Psychiatry* 1999;**66**(1)**:**26-31.

36. Signorini DF, Andrews PJ, Jones PA, et al. Predicting survival using simple clinical variables: a case study in traumatic brain injury. *J Neurol Neurosurg Psychiatry* 1999;**66**(1)**:**20-5.

37. Lai YC, Chen FG, Goh MH, et al.. Predictors of long-term outcome in severe head injury. *Ann Acad Med Singapore* 1998;**27**(3)**:**326-31.

38. Alvarez M, Nava JM, Rue M, Quintana S. Mortality prediction in head trauma patients: performance of Glasgow Coma Score and general severity systems. *Crit Care Med* 1998;**26**(1)**:**142-8.

39. Lang EW, Pitts LH, Damron SL, et al. Outcome after severe head injury: an analysis of prediction based upon comparison of neural network versus logistic regression analysis. *Neurol Res* 1997;**19**(3)**:**274-80.

40. Cho DY, Wang YC. Comparison of the APACHE III, APACHE II and Glasgow Coma Scale in acute head injury for prediction of mortality and functional outcome. *Intensive Care Med* 1997;**23**(1)**:**77-84.

41. Combes P, Fauvage B, Colonna M, et al. Severe head injuries: an outcome prediction and survival analysis. *Intensive Care Med* 1996;**22**(12)**:**1391-5.

42. Zafonte RD, Hammond FM, Mann NR, et al. Revised trauma score: an additive predictor of disability following traumatic brain injury? *Am J Phys Med Rehabil* 1996;**75**(6)**:**456-61.

43. Mamelak AN, Pitts LH, Damron S. Predicting survival from head trauma 24 hours after injury: a practical method with therapeutic implications. *J Trauma* 1996;**41**(1)**:**91-9.

44. Walder AD, Yeoman PM, Turnbull A. The abbreviated injury scale as a predictor of outcome of severe head injury. *Intensive Care Med* 1995;**21**(7)**:**606-9.

45. Cooke RS, McNicholl BP, Byrnes DP. Use of the Injury Severity Score in head injury. *Injury* 1995;**26**(6)**:**399-400.

46. Temkin NR, Holubkov R, Machamer JE, et al. Classification and regression trees (CART) for prediction of function at 1 year following head trauma. *J Neurosurg* 1995;**82**(5)**:**764-71.

47. Fearnside MR, Cook RJ, McDougall P, et al. The Westmead Head Injury Project outcome in severe head injury. A comparative analysis of pre-hospital, clinical and CT variables. *Br J Neurosurg* 1993;**7**(3)**:**267-79.

48. Vilalta J, Vaque J, Olona M, et al. [Predictive factors of mortality in severe craniocerebral trauma]. *Med Clin (Barc)* 1992;**99**(12)**:**441-3.

49. Ross SE, O'Malley KF, Stein S, et al. Abbreviated injury scaling of head injury as a prognostic tool for functional outcome. *Accid Anal Prev* 1992;**24**(2)**:**181-5.

50. Benzer A, Mitterschiffthaler G, Marosi M, et al. Prediction of non-survival after trauma: Innsbruck Coma Scale. *Lancet* 1991;**338**(8773)**:**977-8.

51. Choi SC, Muizelaar JP, Barnes TY, et al. Prediction tree for severely head-injured patients. *J Neurosurg* 1991;**75**(2)**:**251-5.

52. Feldman Z, Contant CF, Robertson CS, et al. Evaluation of the Leeds prognostic score for severe head injury. *Lancet* 1991;**337**(8755)**:**1451-3.

53. Zagara G, Scaravilli P, Mastorgio P, et al. Validation of a prognostic system in severe brain-injured patients. *J Neurosurg Sci* 1991;**35**(2)**:**77-81.
