# Supplementary material for: Systematic review of prognostic models in traumatic brain injury
Source: BMC Med Inform Decis Mak. 2006 Nov 14;6:38. doi: 10.1186/1472-6947-6-38 (PMC1657003; doi:10.1186/1472-6947-6-38)
Supplement: Additional File 4 — General Characteristics of the models. This table describe the characteristics of the included models in the systematic review [file 1472-6947-6-38-S4.pdf]

General characteristics of the models

Study number	Author	Year of publication	Age group	Severity	Objective	Outcome	Multivariable analysis	N° of patients included
1	choi	1991	nr	severe	develop	GOS	CART	555
2	feldman	1991	nr	all	validate	mortality	na	479
2	feldman	1991	nr	all	validate	mortality	na	131
3	benzer	1991	all	severe	validate	mortality	na	421
4	zagara	1991	adults	severe	validate	mortality	na	76
5	vilalta	1992	all	severe	develop	mortality	logistic	173
6	ross	1992	all	severe	validate	GOS	na	503
7	fearnside	1993	all	severe	develop	mortality	logistic	315
7	fearnside	1993	all	severe	develop	functional	logistic	315
8	walder	1995	adults	severe	validate	GOS	na	109
9	temkin	1995	nr	all	develop	functional	CART	448
9	temkin	1995	nr	all	develop	functional	CART	448
9	temkin	1995	nr	all	develop	functional	CART	448
9	temkin	1995	nr	all	develop	functional	CART	448
9	temkin	1995	nr	all	develop	functional	CART	448
9	temkin	1995	nr	all	develop	functional	CART	448
10	cooke	1995	nr	severe	validate	GOS	na	131
11	mamelak	1996	all	severe	develop	GOS	logistic	672
12	combes	1996	nr	severe	develop	GOS	logistic	198
13	zafonte	1996	adults	all	validate	functional	na	501
14	lang	1997	all	severe	develop	mortality	logistic	799
14	lang	1997	all	severe	develop	mortality	logistic	799
14	lang	1997	all	severe	develop	mortality	neural network	799
14	lang	1997	all	severe	develop	mortality	neural network	799
15	cho	1997	adults	all	validate	mortality	na	200
15	cho	1997	adults	all	validate	mortality	na	200
15	cho	1997	adults	all	validate	mortality	na	200
15	cho	1997	adults	all	validate	functional	na	200
15	cho	1997	adults	all	validate	mortality	na	200
15	cho	1997	adults	all	validate	mortality	na	200
15	cho	1997	adults	all	validate	mortality	na	200
15	cho	1997	adults	all	validate	functional	na	200
16	alvarez	1998	adults	all	validate	mortality	na	247
16	alvarez	1998	adults	all	validate	mortality	na	247

Study number	Author	Year of publication	Age group	Severity	Objective	Outcome	Multivariable analysis	N° of patients included
16	alvarez	1998	adults	all	validate	mortality	na	247
16	alvarez	1998	adults	all	validate	mortality	na	247
17	lai	1998	adults	severe	validate	GOS	na	70
18	signorini	1999	adults	severe and moderate	develop	mortality	logistic	110
19	signorini	1999	adults	severe and moderate	develop	mortality	logistic	372
20	nissen	1999	all	all	validate	GOS	na	324
21	sakellaropoulos	1999	nr	nr	develop	GOS	bayesian	525
22	stuss	2000	adults	all	develop	functional	CART	187
22	stuss	2000	adults	all	develop	functional	weibull regression	187
23	sustic	2000	adults	all	develop	mortality	no	41
23	sustic	2000	adults	all	develop	mortality	no	43
24	mukherjee	2000	all	severe	develop	GOS	logistic	103
25	sinha	2000	children	nr	develop	CT scan lesion	neural network	351
25	sinha	2000	children	nr	develop	CT scan lesion	logistic	351
26	ashwal	2000	children	nr	develop	functional	linear discriminant analysis	27
26	ashwal	2000	children	nr	develop	functional	linear discriminant analysis	26
27	lannoo	2000	adults	severe	develop	GOS	logistic	78
27	lannoo	2000	adults	severe	develop	mortality	logistic	158
28	vath	2000	nr	severe	develop	GOS	neural network	95
28	vath	2000	nr	severe	develop	GOS	neural network	95
28	vath	2000	nr	severe	develop	GOS	neural network	95
28	vath	2000	nr	severe	develop	GOS	neural network	95
29	wagner	2000	adults	all	validate	functional	na	378
29	wagner	2000	adults	all	validate	functional	na	378
29	wagner	2000	adults	all	validate	functional	na	378
29	wagner	2000	adults	all	validate	functional	na	378
29	wagner	2000	adults	all	validate	functional	na	378
29	wagner	2000	adults	all	validate	functional	na	378
29	wagner	2000	adults	all	validate	functional	na	378

Study number	Author	Year of publication	Age group	Severity	Objective	Outcome	Multivariable analysis	N° of patients included
29	wagner	2000	adults	all	validate	functional	na	378
30	wagner	2000	adults	all	validate	functional	na	378
30	wagner	2000	adults	all	validate	functional	na	378
31	adachi	2000	all	nr	nr	GOS	linear discriminant analysis	63
32	schaan	2001	all	nr	develop	GOS	not clear	554
33	schreiber	2002	adults	severe	develop	mortality	logistic	368
34	ratanalert	2002	all	severe	develop	GOS	logistic	337
35	andrews	2002	all	all	develop	GOS	CART	124
35	andrews	2002	all	all	develop	GOS	CART	124
35	andrews	2002	all	all	develop	GOS	CART	124
36	heard	2002	children	nr	validate	mortality	na	119
37	pillai	2003	adults	severe	develop	GOS	logistic	289
38	tender	2003	all	mild	develop	GOS	no	255
39	brenner	2003	children	severe	develop	functional	discriminant analysis	22
39	brenner	2003	children	severe	develop	functional	linear discriminant analysis	22
39	brenner	2003	children	severe	develop	functional	discriminant analysis	22
40	cassidi	2003	children	all	develop	functional	logistic	3491
41	bush	2003	adults	all	validate	functional	na	294
42	rovlias	2004	adults	severe	develop	GOS	CART	345
43	ibañez	2004	adults	mild	develop	CT scan lesion	logistic	1101
43	ibañez	2004	adults	mild	develop	CT scan lesion	logistic	1101
43	ibañez	2004	adults	mild	develop	CT scan lesion	CART	1101
43	ibañez	2004	adults	mild	develop	CT scan lesion	CART	1101
44	demetriades	2004	all	severe and moderate	validate	mortality	na	7764
45	fabrri	2004	adults	mild	develop	CT scan lesion	logistic	5578
45	fabrri	2004	adults	mild	develop	neurosurgical intervention	logistic	5578
45	fabrri	2004	adults	mild	develop	GOS	logistic	5578

Study number	Author	Year of publication	Age group	Severity	Objective	Outcome	Multivariable analysis	N° of patients included
46	carter	2005	children	severe	develop	GOS	na	102
47	levin	2005	adults	mild	develop	functional	logistic	129
48	eftekhar	2005	adults	all	develop	mortality	logistic	1271
48	eftekhar	2005	adults	all	develop	mortality	neural network	1271
49	hsu	2005	all	severe and moderate	develop	GOS	neural network	3345
50	wechsler	2005	children	all	develop	functional	logistic	4439
50	wechsler	2005	children	all	develop	functional	logistic	4439
51	poon	2005	nr	severe and moderate	develop	GOS	logistic	68
52	hukkelhoven	2005	nr	severe and moderate	develop	neurosurgical intervention	logistic	275
52	hukkelhoven	2005	nr	severe and moderate	develop	raised IPC	logistic	275
53	hukkelhoven	2005	nr	severe and moderate	develop	GOS	logistic	2269
53	hukkelhoven	2005	nr	severe and moderate	develop	mortality	logistic	2269
